# Anti-Inflammatory and Antioxidant Properties of the Extract, Tiliroside, and Patuletin 3-O-*β*-D-Glucopyranoside from* Pfaffia townsendii* (Amaranthaceae)

**DOI:** 10.1155/2018/6057579

**Published:** 2018-09-30

**Authors:** Wallace Ribeiro Corrêa, Alessandra Freitas Serain, Leticia Aranha Netto, Jane V. N. Marinho, Arielle Cristina Arena, Diana Figueiredo de Santana Aquino, Ângela Midori Kuraoka-Oliveira, Armando Jorge Júnior, Laura Priscila Toledo Bernal, Cândida Aparecida Leite Kassuya, Marcos José Salvador

**Affiliations:** ^1^Institute of Biology, Department of Plant Biology, PPG BTPB and PPGBV, State University of Campinas (UNICAMP), Campinas, SP, Brazil; ^2^Federal Institute of Education, Science and Technology, South of Minas Gerais (IFSULDEMINAS), 37576000 Inconfidentes, MG, Brazil; ^3^School of Health Sciences, Federal University of Grande Dourados (UFGD), Dourados, MS, Brazil; ^4^School of Health Sciences, University Center of Grande Dourados (UNIGRAN), Dourados, MS, Brazil; ^5^University Hospital (HU-UFGD), Federal University of Grande Dourados (UFGD), Dourados, MS, Brazil

## Abstract

Brazilian ginseng, including* Pfaffia townsendii*, is used in popular medicine as a natural anti-inflammatory, tonic, analgesic, and antidiabetic agent. In this study, we investigated the chemical composition and evaluated the antioxidant and anti-inflammatory activities of the* P. townsendii* ethanolic extract as well as the major isolated glycoside flavonoids tiliroside and patuletin 3-O-*β*-D-glucopyranoside. Chromatographic techniques and spectroscopic analysis were used for the isolation and identification of the major compounds. The antioxidant potential was determined through DPPH and ORAC-FL assays. The total phenolic content was measured using Folin-Ciocalteu reagent. The anti-inflammatory activity was determined based on a model of paw edema and carrageenan- (Cg-) induced pleurisy. We identified three phenolic acids, one carboxylic acid and two flavonoids, patuletin 3-O-*β*-D-glucopyranoside, and tiliroside. The ethanol crude extracts, partitions and isolated flavonoids (4581 *μ*mol of Trolox equivalents/g of extract in ORAC and a SC_50_ of approximately 31.9 *μ*g/mL in the DPPH assay) demonstrated antioxidant activity, and the ethanolic extract as well as isolated flavonoids inhibited paw edema induced by Cg and leukocyte migration in the Cg-induced pleurisy model. The extract, tiliroside, and patuletin 3-O-*β*-D-glucopyranoside obtained from* P. townsendii* have therapeutic potential against oxidative stress-related and inflammatory disorders.

## 1. Introduction

In Brazil, species of the genus* Pfaffia* (Amaranthaceae) are sold in the market as substitutes for* Panax *spp. (Ginseng, Araliaceae) because their roots are very similar. The population uses the popular “Brazilian ginseng” because of its anti-inflammatory, antistress [1], aphrodisiac, tonic, analgesic, and antidiabetic properties [2, 3]. The species acts against inflammation [1], cancer, gastric disorders, and rheumatism [3, 4] and is used to prevent premature aging, colic, and enteritis [5, 6].

Pharmacological studies have demonstrated that* Pfaffia* species are promising for the development of new molecules or therapies because they contain compounds with anti-inflammatory and analgesic activities [7, 8], as well as compounds that protect the gastric mucosa against lesions [4] and that have antioxidant [9] and antineoplastic activities [10].

Studies have shown that species of the Amaranthaceae family are able to accumulate phenolic substances, which have antioxidant activity, scavenge free radicals, protect against lipid peroxidation, and quench reactive oxygen species [11, 12]. Oxidative stress is present in some diseases such as cancers [13], cardiovascular disorders [14], inflammatory arthritis [15], and Alzheimer's disease [16].

Glycoside flavonoids, such as tiliroside and patuletin 3-O-*β*-D-glucopyranoside, are found in several plants and are interesting because they could be responsible for the medicinal activity of these plants. For example, tiliroside has the ability to inhibit iNOX and COX-2 expression as well as MAPK/JNK/p38-mediated inflammation in LPS-stimulated macrophages* in vitro* [17]. Velagapud et al. (2014) also showed that tiliroside has* in vitro* inhibitory activity in microglia during neuroinflammation through a mechanism involving the TRAF-6-mdiated activation of the NF-*κ*B and p38 MAPK signaling pathways [18]. This compound has been shown to exhibit anti-inflammatory activity in a TPA model and against phospholipase A2-induced edema and to have antioxidant and hepatoprotective activities [19]. Patuletin and its derivatives also show biological activity. Jabeen et al. (2016) revealed the anti-inflammatory and antiarthritic activities of patuletin in rodent models [20], whereas Li et al. (1991) showed that patuletin inhibits rat lens aldose reductase [21]. Additionally, patuletin acetylrhamnoside derivatives from* Kalanchoe brasiliensis* act as inhibitors of human lymphocyte proliferative activity.

All these facts prompted us to investigate the chemical composition and evaluate the antioxidant and anti-inflammatory activities of the* Pfaffia townsendii* ethanolic extract (EEPT) and its major isolated glycoside flavonoids tiliroside and patuletin 3-O-*β*-D-glucopyranoside.

## 2. Materials and Methods

### 2.1. Plant Material

Plants of* P. townsendii* were collected at Pico das Almas (Bahia, Brazil) and identified by Professor Josafá Carlos de Siqueira (Pontificia Universidade Catolica, Rio de Janeiro). A voucher specimen was deposited at the Herbarium at the Faculty of Philosophy, Sciences and Letters of Ribeirão Preto- (FFCLRP-) USP under register number NPL396.

### 2.2. Extraction and Isolation of Compounds

Whole* P. townsendii* plants were dried in an open stove at 40°C and powdered, affording 100 g. Exhaustive extraction with ethanol after hexane extraction gave 9.8 g of the hexane crude extract and 38 g of the ethanol crude extract (dry weight). The ethanol extract of* P. townsendii *(EEPT, 35 g) was suspended in MeOH/H_2_O (9:1, v/v) and partitioned with hexane, resulting in hexanic phase after removal of the solvent in a rotary evaporator (2 g). To the remaining hydroalcoholic phase water was added up to 40% and extracted with dichloromethane, resulting in the dichloromethane phase after removal of the solvent in a rotary evaporator (11 g). The remaining hydroalcoholic phase after removal of the solvent in a rotary evaporator, followed by the lyophilization (20 g), was subjected to fractionation using column chromatography with a Sephadex LH-20 stationary phase and MeOH mobile phase. Sixty-eight fractions were obtained after TLC analysis (using silica gel plates and butanol/acetic acid/water (65:15:25, v/v/v) as the mobile phase) and were pooled into 20 fractions. In fraction 8 (115 mg) and fractions 13-18 (130 mg) yellow precipitate was observed, that, after washing with cold ether, resulted, respectively, in isolated flavonoids patuletin 3-*O*-*β*-D-glucopyranoside (yield = 70 mg) and tiliroside (yield = 110 mg). These isolated compounds were identified by spectroscopic analyses (IR, UV, ^1^H NMR, ^13^C NMR, Dept. 135°, and HMBC) and ESI-MS/MS and comparison with the literature data [22–25].

### 2.3. HPLC-DAD and ESI-MS Analyses

The EEPT and partitions were diluted in a solution containing 50% (v/v) chromatography-grade methanol, 50% (v/v) deionized water, and 0.5% ammonium hydroxide. In the fingerprinting ESI-MS analysis, the general conditions were as follows: source temperature, 100°C; capillary voltage, 3.0 kV; and cone voltage, 30 V. For measurements in the negative-ion mode, ESI(-)-MS, 10.0 *μ*L of concentrated NH_4_OH was added to the sample mixture to obtain a total volume of 1000 *μ*L with a final concentration of 0.1%. ESI-MS was performed by direct infusion with a flow rate of 10 *μ*L min mL^−1^ using a syringe pump. Structural analysis of single ions in the mass spectrum of each extract was performed by ESI-MS/MS. The ion with the* m/z* of interest was selected and submitted to 15–45 eV collisions with argon in the collision quadrupole. The compounds were identified by comparison of their ESI-MS/MS fragmentation spectra with standard samples [26–28]. HPLC analyses were conducted using an RP-18 column (Lichrospher, 5 *μ*m, 225∖4.6 mm, Merck). The mobile phase consisted of a linear gradient combining solvent A (acetonitrile) and solvent B (water/acetic acid, 99:1, v/v, pH = 2.88) as follows: 15% A (15 min), 15–20% A (7 min), 20% A (5 min), 20–40% A (5 min), 40% A (5 min), and 40–15% A (3 min). The analyses were performed in triplicate at a flow rate of 0.8 mL/min and with an injection volume of 20 *μ*L. The UV-DAD detector was set to record between 200 and 600 nm, and the UV chromatograms were measured at 254 and 330 nm. Quantitative HPLC-ESI-MS analyses were performed on the EEPT to verify the contents of the isolated flavonoids patuletin 3-O-*β*-D-glucopyranoside and tiliroside in the sample.

### 2.4. Quantitative Determination of the Total Soluble Phenols

The extract and partitions dissolved in methanol were analyzed for their total soluble phenolic content according to the Folin-Ciocalteu colorimetric method [29, 30] using gallic acid as a reference. The results are expressed as mg of gallic acid equivalents (GAE) per gram of extract or fraction (mg of GAE/g). The analyses were performed in triplicate.

### 2.5. Assessment of Radical Scavenging Activity Using the DPPH Method

The antiradical activities of the extracts were determined using the stable 2,2-diphenyl-1-picrylhydrazyl (DPPH) radical [31]. The test was performed in 96-well microplates. Fifty microliters of a 250 *μ*M DPPH solution in MeOH was added to a range of solutions of different concentrations (7 serial 3-fold dilutions to give a final range of 100 to 1.6 *μ*g mL^−1^) of extracts to be tested in MeOH (10 *μ*L). The absorbance at 517 nm was measured 30 min after the addition of each of the tested compounds, and the percentage of activity was calculated. Quercetin and 6-hydroxy-2,5,7,8-tetramethylchroman-2-carboxylic acid (Trolox) were used as positive controls. All the samples were tested in triplicate. The antioxidant activity of each sample is expressed as the SC_50_ value, which is the concentration in *μ*g mL^−1^ of each extract that scavenged 50% of the DPPH radicals. All the results are expressed as the mean of three different trials.

### 2.6. Evaluation of the Antioxidant Capacity by the ORAC Assay

The antioxidant capacity of the ethanolic extract was assessed by the oxygen radical absorbance capacity (ORAC) kinetic assay according to the method established by Ou and coworkers [32, 33] with modifications [12]. The data are expressed as micromoles of 6-hydroxy-2,5,7,8-tetramethylchroman-2-carboxylic acid (Trolox) equivalents (TE) per gram of extract on a dry weight basis (*μ*mol of TE/g) and as relative Trolox equivalents for pure compounds. Quercetin, vitexin, caffeic acid, and chlorogenic acid were used as positive controls. The analyses were performed in triplicate.

### 2.7. Animals

For the inflammation test,* Swiss* male (edema test) or female (pleurisy test) mice (25–35 g) were used. The animals were maintained under a 12 h light–dark cycle with a controlled humidity of 60–80% and temperature of 22±1°C. Food and water were administered* ad libitum*, and approved experimental protocols were followed (number 020/2014 by the Ethics Committee for Research on Laboratory Animal Use of the UFGD).

### 2.8. Paw Edema Model of Cg to Induce Inflammation

Male* Swiss* mice were treated with an oral dose of the EEPT (300 mg/kg), patuletin 3-O-*β*-D-glucopyranoside (1 mg/kg), tiliroside (1 mg/kg), and vehicle (control), which was administered 60 minutes before Cg. Cg (300 *μ*g) was administered by a subcutaneous route in the right paw of 100 *μ*L of a solution prepared with 0.9% saline to the groups cited before as well as the control group (treated only with vehicle, 0.9% saline) and the positive control group (treated with subcutaneous (s.c.) dexamethasone, 1 mg/kg). The left paw was injected with 100 *μ*L of 0.9% saline. The measurements of paw volume changes were made with a digital water plethysmometer (from Panlab) at 1, 2, and 4 h after Cg injection [34].

### 2.9. Induction of Pleurisy

The same doses of the EEPT, patuletin 3-O-*β*-D-glucopyranoside, and tiliroside described in Section 2.8 were also used to treat different groups (three groups with n = 6) of female* Swiss* mice via an oral route (p.o.), whereas the control group received 0.9% saline (p.o.). The naïve group received 0.9% saline (p.o.) but did not receive Cg via an intrathoracic route (i.t.), and the last group received dexamethasone (1 mg/kg positive control) via the s.c. route [35]. An i.t. injection of a solution of 1% Cg (100 *μ*L) and sterile saline (100 *μ*L; only to the naïve mice) was administered, and after 4 h, the animals were killed. The thorax was opened to obtain the pleural exudate [36]. After lavage, an aliquot of 50 *μ*L was diluted with Evans blue dye (1:20) and used to determine the total number of leukocytes in a Neubauer chamber. The protein content in the extravasated fluid was assessed by the Bradford method (Bioagency, Brazil).

### 2.10. Statistical Analysis

The data are reported as the means (%RSDs, relative standard deviations) of triplicate determinations. The statistical analyses were carried out using Microsoft Excel 2013 (Microsoft Corp., Redmond, WA, USA) and GraphPad Prism version 5.0 for Windows (GraphPad Software, San Diego, CA, USA). The data are presented as the means ± SEM. Differences between groups were evaluated by analysis of variance (one-way ANOVA) followed by the Newman–Keuls test. The number of animals per group is indicated in the figure legends. Statistical differences were considered significant at P < 0.05. Asterisks (*∗*) denote a significant difference compared with the vehicle-treated group.

## 3. Results

### 3.1. Isolation of Flavonoids and Phytochemical Analysis

**Figure 1:**
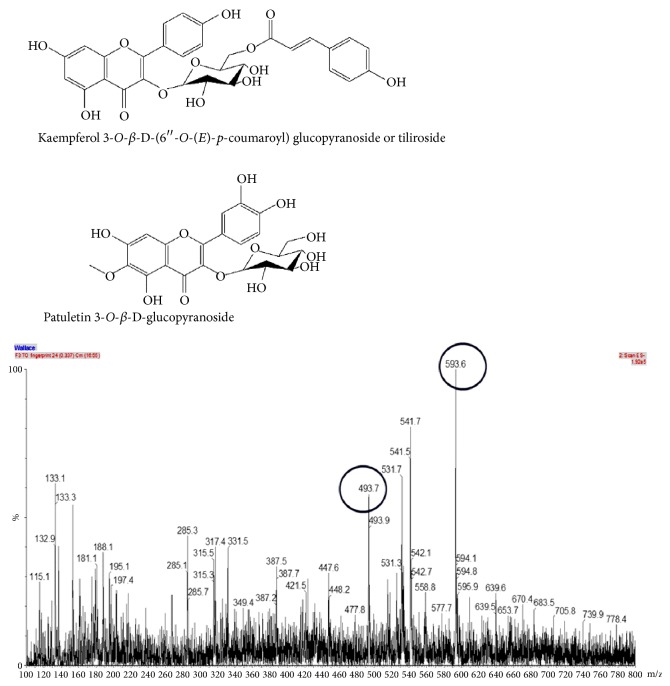
ESI-MS spectra of ethanol extract and chemical structure of flavonoids identified and isolated from* Pfaffia townsendii*.

The ethanolic extract of* P. townsendii* was subjected to fractionation, and a phytochemical investigation of this extract led to the isolation of two flavonoids, patuletin 3-*O*-*β*-D-glucopyranoside and kaempferol 3-*O*-*β*-D-(6′′-*O*-(*E*)-*p*-coumaroyl) glucopyranoside or tiliroside (Figure 1).

This manuscript constitutes the first report of these compounds in* P. townsendii*. The three phenolic acids, one carboxylic acid and two flavonoids (Table 1), were detected by comparisons of their ESI-MS/MS fragmentation spectra with standard samples.

### 3.2. Quantitative HPLC-MS Analysis of the Flavonoids

In the HPLC-MS analysis, the calibration curves of the isolated flavonoids patuletin and tiliroside were obtained using concentrations between 500 *μ*g/mL and 5 *μ*g/ml. The concentrations of these compounds in the EPPT were then calculated based on these curves. The sample showed patuletin 3-O-*β*-D-glucopyranoside and tiliroside contents of 2.45 *μ*g/mg of extract (equation of the line, 13828.8x + 498331.0, R^2^ = 0.9955) and 4.74 *μ*g/mg of extract (equation of the line, y = 21147.4x + 626421.6, R^2^ = 0.9920), respectively.

### 3.3. Antioxidant Analysis

The extracts showed antioxidant activity in DPPH assays, with SC_50_ values varying from 31.9 to 62.6 *μ*g/mL. The crude hexane and the hexane phase partition yielded very low values, with inhibitory concentrations > 200. In DPPH assays, the highest antioxidant activity was exhibited by the ethanolic extract (SC_50_ = 31.9 *μ*g/mL). Moreover, in the ORAC-FL kinetic assay, the extracts showed antioxidant capacity values between 10.0 and 4581 *μ*mol of Trolox equivalents per gram of extract (*μ*mol of TE/g), confirming the low antioxidant activity of the hexane extract and the hexane phase (Table 2). The total phenolic content was measured using the Folin-Ciocalteu reagent. The extracts showed good levels of phenolic compounds (3.3–2.0 mg GAE/g), justifying the antioxidant activity identified through both methods. The ethanolic extract and hydroalcoholic phase of the partition showed excellent results in the antioxidant assays. The antioxidant activities of the two flavonoids were evaluated by DPPH and ORAC assays, verifying that these isolated compounds also showed antioxidant activities (Table 2).

### 3.4. Effect of the Ethanol Extract of the EEPT and Isolated Flavonoids on Cg-Induced Paw Edema

**Figure 2:**
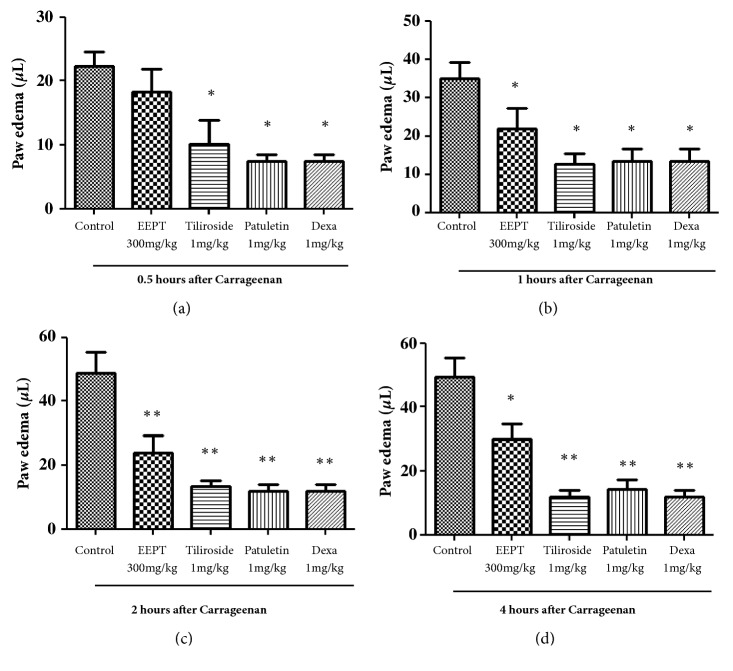
Effect of ethanolic extract of* Pfaffia townsendii* (EEPT) and isolated flavonoids on carrageenan-induced paw edema in mice. Animals received ethanol extract, EEPT, (300 mg/kg, p.o), tiliroside (1.0 mg/kg), patuletin 3-*O*-*β*-D-glucopyranoside (1.0 mg/kg), and dexamethasone (Dexa – 1.0 mg/kg, s.c.) or control (vehicle) and after 0.5 (a), 1 (b), 2 (c), and 4 hours (d), an intraplantar injection of carrageenan (300*μ*g/paw) was performed. Each bar or point represents ± SEM of 6 animals. *∗*P < 0.05, *∗∗*P < 0.01, and *∗∗∗*P < 0.001, compared with vehicle-treated group.

In the paw inflammatory model, a dose of 300 mg/kg of the EEPT reduced the paw volume by 2 h (with a maximal inhibition of 51.00 ± 11.0% compared to the control), as observed 4 h after the Cg injection (Figure 2). Moreover, our results showed that a dose of 1.0 mg/kg of the flavonoids yielded maximal inhibition 240 min after the administration of tiliroside (73.00 ± 4.0%, P < 0.001) and patuletin 3-O-*β*-D-glucopyranoside (75.4 ± 4.0%, P < 0.001) compared with the control, as indicated by the time course analysis (Figure 2(c)). As expected, the inflammatory parameter was significantly reduced in the Cg-induced inflammatory process by treatment with dexamethasone.

### 3.5. Effect of the Ethanol Extract of the EEPT and Isolated Flavonoids on Cg-Induced Leukocyte Migration into the Pleura

The animals treated with the EEPT at a dose of 300 mg/kg, tiliroside (1.0 mg/kg), and patuletin 3-O-*β*-D-glucopyranoside (1.0 mg/kg) showed significant reductions in leukocyte migration induced by Cg in the pleural exudate (Figure 2(a)).

**Figure 3:**
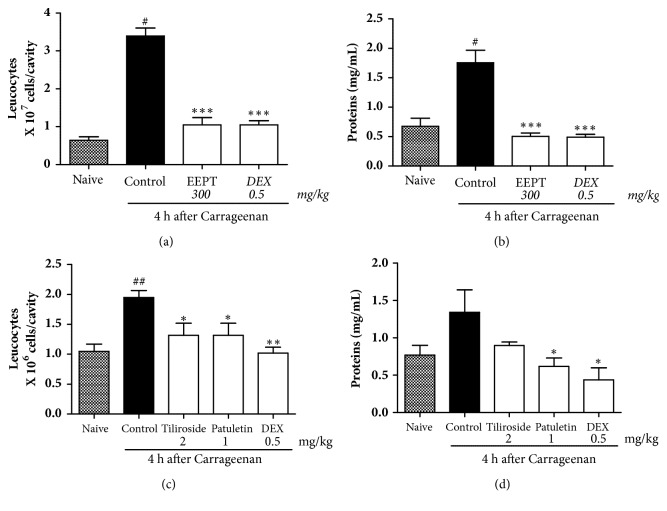
Effect of ethanol extract EEPT of* P. townsendii* (300 mg/kg), tiliroside (1.0 mg/kg), and patuletin 3-*O*-*β*-D-glucopyranoside (1.0 mg/kg) on carrageenan-induced leukocyte migration and plasma leakage into the air pouch. Mice received substances, control, or vehicle 1 h before carrageenan stimulus (n = 6 animals per group). *∗*P < 0.05, *∗∗*P < 0.01, and *∗∗∗*P < 0.001, compared with vehicle-treated group.

The EEPT showed an inhibition of 69.2 ± 1.04% compared with the vehicle group (Figure 3(a)). The naïve group, as expected, did not demonstrate inflammatory parameters in the analysis. The animals that were pretreated with the EEPT showed a plasma leakage of 72.0 ± 0.49%, which is higher than that observed in the animals pretreated with the vehicle (Figure 3(b)). As expected, the number of leukocytes and amount of protein in the pleural exudate were significantly reduced by treatment with dexamethasone.

The isolated flavonoids (patuletin 3-O-*β*-D-glucopyranoside and tiliroside) significantly reduced leukocyte migration (mainly polymorphonuclear cells) to the cavity, as illustrated in Figure 3(c), by 50.7 ± 1.03% and 59.4 ± 1.25%, respectively, compared with the vehicle group. In addition, the animals treated with patuletin 3-O-*β*-D-glucopyranoside and tiliroside showed decreased plasma leakage values of 13.3 ± 0.65% and 40.0 ± 0.46%, respectively, compared with those pretreated with the vehicle (Figure 3(d)).

## 4. Discussion

The present study provides the first demonstration of the antioxidant and anti-inflammatory properties of* P. townsendii*. These properties could be related to the four phenolic acids and two flavonoids found in our phytochemical studies. Both properties were positively verified by testing patuletin 3-O-*β*-D-glucopyranoside and tiliroside individually under the same conditions as the EEPT, indicating that the two flavonoids are important to* P. townsendii *and may contribute to the validation of the folk medicinal uses of Brazilian ginseng, such as anti-inflammatory, analgesic, and antidiabetic uses [3, 4].

The identification of the phenolic acids, carboxylic acid, and flavonoids in* P. townsendii* is in agreement with the literature regarding the Amaranthaceae family [10, 12]. The presence of the flavonoids tiliroside and patuletin 3-O-*β*-D-glucopyranoside in the Amaranthaceae family is also well known, and other studies have identified such glycosylated flavonoids [37, 38]. However, this is the first time that these flavonoids have been identified in* P. townsendii*.

The study showed that the EEPT extract from* P. townsendii* (Table 2) exhibit excellent antioxidant activity. In accordance with the literature, samples with values ≥1000.0 *μ*mol of TE/g can be considered to have good antioxidant capacity [32, 33]. The dichloromethane phase (5641 *μ*mol of TE/g) and hydroalcoholic phase (4581 *μ*mol of TE/g) of partition meet this criterion. The hydroalcoholic phase as fractionated and gave rise to the patuletin 3-O-*β*-D-glucopyranoside and tiliroside flavonoids. The DPPH and ORAC antioxidant analyses of the isolated flavonoids showed excellent antioxidant results, a fact that justifies future biological surveys of* P. townsendii* because the antioxidant capacity of flavonoids confers a preventive and therapeutic potential in cardiovascular diseases [39], cancer [40, 41], and bacterial infections [42]. Sala et al. showed the antioxidant properties of tiliroside in enzymatic lipid peroxidation, nonenzymatic lipid peroxidation, scavenging, and the DPPH test (high potency) [19]. Their antiviral actions [43] as well as antiallergic and anti-inflammatory properties [44] are also important. The increase in the interest in flavonoids as a health benefit has increased due to their potential activity against free radicals and antioxidant power in* in vitro* analyses because increases in free radicals are related to some types of diseases [14–16].

The ethanolic extract of* P. townsendii* inhibited edema formation and protein extravasation and had an inhibitory effect on leukocyte recruitment induced by Cg, which suggests that the fraction possesses compounds that are potentially anti-inflammatory. This was confirmed by the anti-inflammatory evaluation of isolated flavonoids from the ethanolic extract, tiliroside, and patuletin 3-O-*β*-D-glucopyranoside (Figure 2). One study demonstrated the inhibition of the expression of iNOX, and COX-2 as well as the MAPK/JNK/p38-mediated inflammatory process* in vivo* in LPS-stimulated macrophages [17], showing the* in vivo* anti-inflammatory activity of tiliroside in mice. Some studies have shown that tiliroside has anti-inflammatory activity [17–19]. In an* in vivo* inflammatory assay, mouse paw edema induced by phospholipase A was inhibited by tyrosine with an activity at 35.6 mg/kg [19]. Jabeen et al. [20] indicated the anti-inflammatory and antiarthritic activities of patuletin 3-O-*β*-D-glucopyranoside in rodent models. The present work confirmed the efficacy of tiliroside and showed the high potency of this compound in the inhibition of the recruitment of additional immune cells, capillary leakage (indirectly verified by the protein dosage in pleura), and edema. The presence of patuletin 3-O-*β*-D-glucopyranoside and tiliroside contributes to the anti-inflammatory effects of* P. townsendii*.

The anti-inflammatory effect of patuletin 3-O-*β*-D-glucopyranoside is very similar to that of the dexamethasone control (Figures 2 and 3(c)): the flavonoid significantly reduces the increase in vascular permeability and the entrance of immune cells into tissues at the site of an inflammatory reaction (Figure 3(d)). These results confirmed previous observations showing that patuletin 3-O-*β*-D-glucopyranoside significantly inhibits histamine-induced hind-paw edema [45]. The results confirm that patuletin 3-O-*β*-D-glucopyranoside is effective in the treatment of acute inflammation by suppressing the chemical mediators of inflammation. This activity was previously indicated for flavonoids based on the inhibition of inflammation in experimental models through inhibition of the expression of inducible nitric oxide synthase (iNOS), cyclooxygenase (COX), and lipoxygenase (LOX) and subsequent decreases in nitric oxide (NO), prostanoids, leukotrienes, and other mediators of the inflammatory process, such as cytokines, chemokines, or adhesion molecules [46–48].

These results suggest that the anti-inflammatory effects of* P. townsendii* are related to the presence of the flavonoids patuletin 3-*O*-*β*-D-glucopyranoside and tiliroside (Supplementary Material (available here)).

## 5. Conclusion

The results of the present study justify the popular use of* Pfaffia* because extracts of* P. townsendii* showed excellent antioxidant activity, as demonstrated by DPPH radical scavenging activity and ORAC assays. The study also found that the extract shows notable anti-inflammatory activity* in vivo*, and this activity is associated with the presence of the flavonoids patuletin 3-*O*-*β*-D-glucopyranoside and tiliroside. To the best of our knowledge, this report constitutes the first demonstration that the extract of* P. townsendii* has remarkable pharmacological activities related to the presence of flavonoids. Thus,* P. townsendii *may be considered a promising source of antioxidant and anti-inflammatory compounds such as phenolic acids and flavonoids.

## Figures and Tables

**Table 1:** Compounds identified in ethanol extract from *Pfaffia townsendii* using ESI (-)-MS/MS and ESI(+)-MS analyses.

Compound	Deprotonated ions [M-H]^−^*m/z*	MS/MS ions *m/z*
Malic acid	133	15 eV: 133→115
Caffeic acid	179	15 eV: 179→125
Quinic acid	191	25 eV: 191→173, 127, 111, 93, 85
Ferulic acid	195	15 eV: 193→178, 149, 134
Patuletin 3-*O*-*β*-D-glucopyranoside	493	15 eV: 493
Tiliroside	594	15 eV: 594→308, 288

**Table 2:** Total phenol content and antioxidant capacity by the DPPH and ORAC assays of crude extract, phases of partition, and flavonoids isolated from *P. townsendii*.

Sample Ethanol extract	Phenol content^a,b^ (mg of GAE/g)^b^	DPPH assay, SC_50_^a,c^ (*µ*g/mL)^c^	ORAC assay^a,d^ (*µ*mol of TE/g)^d^
Hexane crude extract	< 1.13	> 200	13.0 (5.0)
Ethanol crude extract	3.3 (6.3)	62.6 (10.0)	1555 (10.5)
Hexane phase	1.0 (3.5)	> 200	2461 (9.7)
Dichloromethane phase	2.5 (7.9)	45.6 (6.3)	5641 (4.9)
Hydroalcoholic phase	2.0 (2.8)	31.9 (7.7)	4581 (7.7)

Patuletin 3-*O*-*β*-D-glucopyranoside	-	4.9 (2.1)	4.2 (6.9)^e^

Tiliroside	-	83.2 (3.2)	0.8 (3.4)^e^

Mixture of patuletin 3-*O*-*β*-D-glucopyranoside + Tiliroside		3.7 (2.5)	4.8 (11.4)^e^

Quercetin*∗*	-	8.3 (2.1)	5.6 (0.9)^e^
Caffeic acid*∗*	-	11.2 (2.4)	2.9 (2.0)^e^
Trolox*∗*	-	2.8 (1.8)	-

^a^Mean value (%RSD, relative standard deviation) of triplicate assays. ^b^Total phenolics data expressed as mg of gallic acid equivalents per g (mg of GAE/g). ^c^DPPH assay data expressed as SC_50_ (concentration that inhibited 50% of the DPPH radical) in *μ*g per mL. ^d^ORAC data expressed as *μ*mol of Trolox equivalents per g (*µ*mol of TE/g). ^e^ORAC data expressed as relative Trolox equivalent, mean (%RSD, relative standard deviation) of triplicate assays. *∗*Experimental positive controls: not evaluated.

## Data Availability

The data used to support the findings of this study are available from the corresponding author upon request.

## Supplementary Materials

Supplementary Materials The Supplementary Materials Description: “The* P. townsendii* shows notable anti-inflammatory activity in vivo, and this activity is associated with the presence of the flavonoids patuletin 3-O-*β*-D-glucopyranoside and tiliroside”.
