# Supplementary figures and images for: Anti-Inflammatory and Antioxidant Properties of the Extract, Tiliroside, and Patuletin 3-O-β-D-Glucopyranoside from Pfaffia townsendii (Amaranthaceae)
Source: Evid Based Complement Alternat Med. 2018 Sep 30;2018:6057579. doi: 10.1155/2018/6057579 (PMC6186378; doi:10.1155/2018/6057579)

# Anti-inflammatory and Antioxidant Effects of *Pfaffia townsendii*

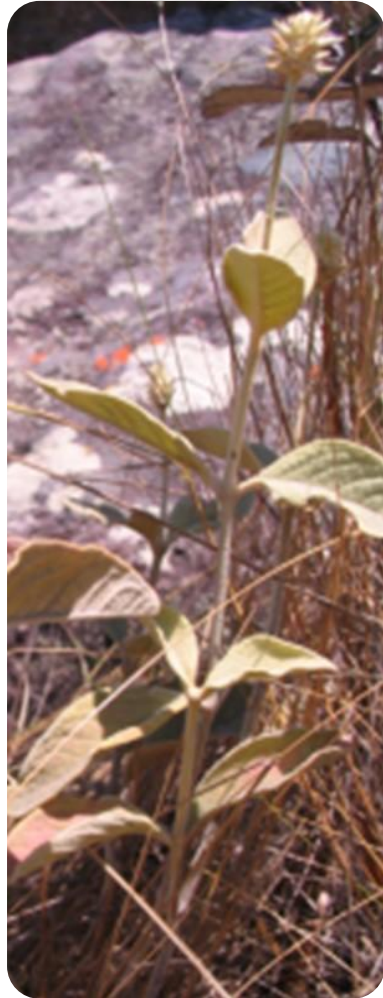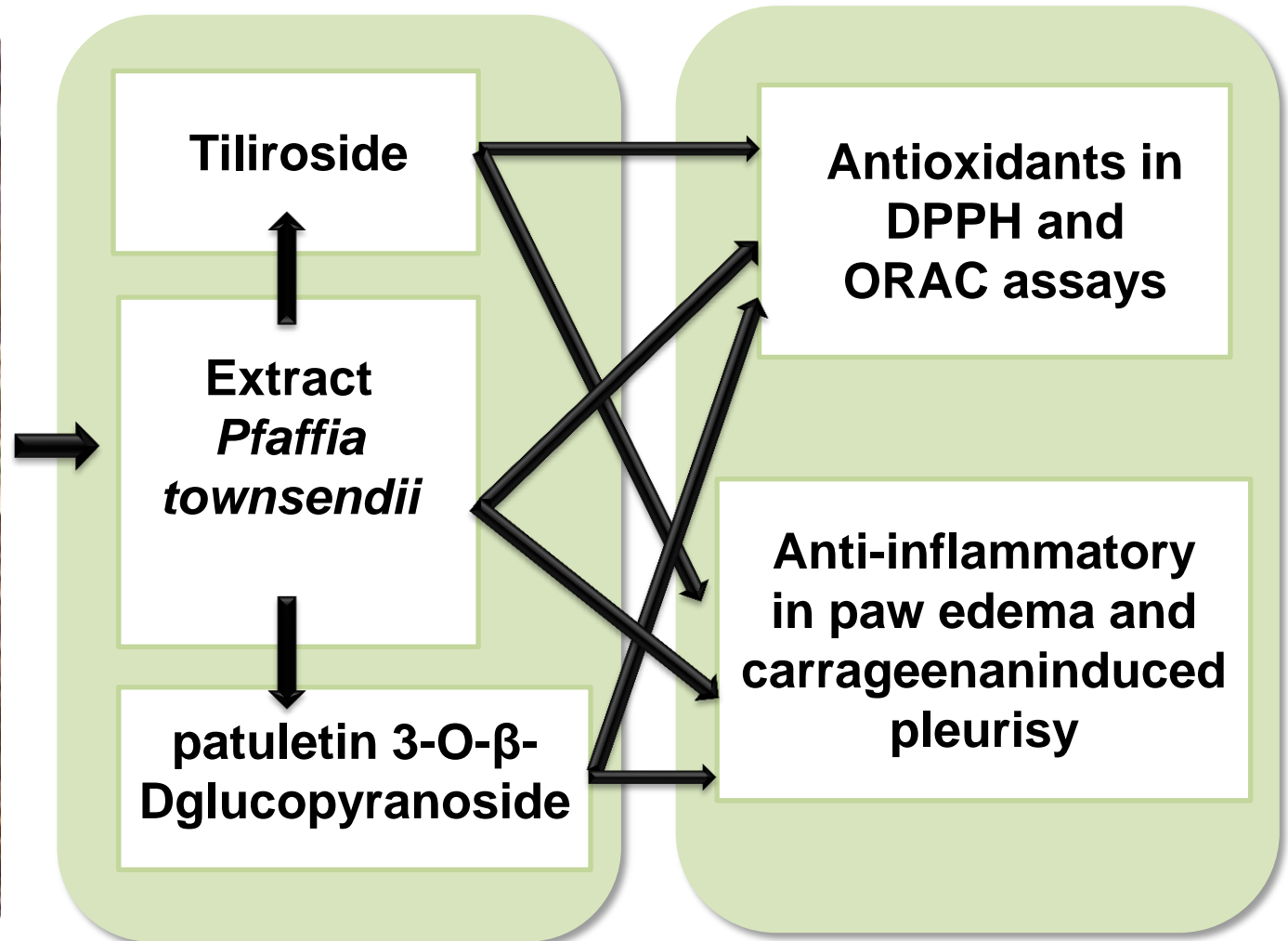

Supplement: Supplementary Materials — The Supplementary Materials Description: “The P. townsendii shows notable anti-inflammatory activity in vivo, and this activity is associated with the presence of the flavonoids patuletin 3-O-β-D-glucopyranoside and tiliroside”. [file 6057579.f1.pdf]
